# Resistance to Dutch Elm Disease Reduces Presence of Xylem Endophytic Fungi in Elms (*Ulmus* spp.)

**DOI:** 10.1371/journal.pone.0056987

**Published:** 2013-02-28

**Authors:** Juan A. Martín, Johanna Witzell, Kathrin Blumenstein, Elzbieta Rozpedowska, Marjo Helander, Thomas N. Sieber, Luis Gil

**Affiliations:** 1 Departamento de Silvopascicultura, Escuela Técnica Superior de Ingenieros de Montes, Universidad Politécnica de Madrid, Madrid, Spain; 2 Southern Swedish Forest Research Centre, Faculty of Forest Sciences, Swedish University of Agricultural Sciences, Alnarp, Sweden; 3 Chemical Ecology, Swedish University of Agricultural Sciences, Alnarp, Sweden; 4 Department of Biology, Section of Ecology, University of Turku, Turku, Finland; 5 Institute of Integrative Biology, Eidgenössische Technische Hochschule (ETH) Zurich, Zurich, Switzerland; Texas A&M University, United States of America

## Abstract

Efforts to introduce pathogen resistance into landscape tree species by breeding may have unintended consequences for fungal diversity. To address this issue, we compared the frequency and diversity of endophytic fungi and defensive phenolic metabolites in elm (*Ulmus* spp.) trees with genotypes known to differ in resistance to Dutch elm disease. Our results indicate that resistant *U. minor* and *U. pumila* genotypes exhibit a lower frequency and diversity of fungal endophytes in the xylem than susceptible *U. minor* genotypes. However, resistant and susceptible genotypes showed a similar frequency and diversity of endophytes in the leaves and bark. The resistant and susceptible genotypes could be discriminated on the basis of the phenolic profile of the xylem, but not on basis of phenolics in the leaves or bark. As the Dutch elm disease pathogen develops within xylem tissues, the defensive chemistry of resistant elm genotypes thus appears to be one of the factors that may limit colonization by both the pathogen and endophytes. We discuss a potential trade-off between the benefits of breeding resistance into tree species, versus concomitant losses of fungal endophytes and the ecosystem services they provide.

## Introduction

Fungal communities play key roles in global carbon sequestration and nutrient mineralization [1] and, for example, the importance of mycorrhizal symbionts for the growth of forest trees has been long established. A less well characterized group of fungal symbionts of forest trees are the endophytic fungi that live at least part of their lives within the aerial tissues of their hosts without causing symptoms [2], [3]. Over time, and with conditioning from host-intrinsic and environmental factors, the nature of the tree-endophyte interaction can change and there is a continuum, ranging from neutral association to mutualistic, pathogenic or saprotrophic interactions [4]–[6]. Given suitable conditions, certain fungi can adopt any one of these life-styles [7], adding a further dimension of functional complexity to this layer of biodiversity inside plants.

Endophytes may provide their host plants with an epigenetic mechanism of adaptation to environmental stress [8], [9]. Moreover, some fungal endophytes seem to protect plants against pathogens [10] and herbivores [11], [12]. As primary colonizers some endophytes can be actively involved in the degradation of dead tissues [13], [14]. Endophytic fungi may thus significantly contribute to the support and regulation of ecosystem services in forests. However, we still lack basic knowledge about regulation and functions of endophytic communities in forest ecosystems. For instance, it is not known whether the resistance status of a tree genotype against aggressive pathogens affects the establishment of endophytic fungi within it. This is a crucial issue for sound evaluation of the goals and approaches applied in forest conservation, restoration and tree breeding because resistance may then have environmental trade-off effects, potentially cascading from individuals to trophic levels and communities. Thus, alterations in endophytic communities in resistant trees could lead to modifications of ecosystem services (e.g. nutrient cycling) (cf. [15]).

In order to explore the possible trade-off between disease resistance and endophyte diversity in forest trees, it is necessary to study the endophytic communities in tree genotypes that express basal resistance or susceptibility to an aggressive pathogen. Elms (*Ulmus* spp.) are forest and amenity trees that are severely affected by the Dutch elm disease (DED) pathogen, *Ophiostoma novo-ulmi* Brasier, and they provide a suitable model system to study the links between pathogen resistance and endophyte colonization in forest trees. *Ulmus minor* Mill., the main elm species studied in this work, has usually a dominant role in riparian forests of southern Europe. The vascular pathogen *O. novo-ulmi* is introduced into healthy elms by elm bark beetles and moves within xylem tissues, ultimately resulting in the development of a wilt syndrome [16]. DED has killed over 1 billion elm trees in Europe and North America. To assist attempts to conserve elm genetic resources, elm genotypes exhibiting high or low susceptibility to DED have been selected and are maintained as clones [17], [18]. This material allows detailed investigations of factors, such as the endophytic flora, that contribute to the phenotypic resistance of elms to DED.

The basal resistance of elms to DED does not follow a major-gene pattern, but is polygenic (quantitative) in nature [19], and the traits behind this type of resistance are still poorly understood. One polygenic trait potentially contributing to plants’ resistance to pathogens are phenolic compounds, defensive and signalling metabolites [20], [21]. Their involvement in DED-induced responses has been demonstrated [22]–[24], but the role of constitutively expressed phenolics in the DED-resistance of elms is still unclear, and we do not know if the endophytic communities in elms are affected by them. Furthermore, other polygenic traits may be important for the DED resistance. In comparison to major-gene resistance, polygenic resistance is often considered more durable [25] and thus appears to be an attractive goal for resistance breeding. However, the drawbacks of quantitative resistance include the necessity of vegetative propagation [26], which could lead to a risk of low genetic variability in the propagated population. Moreover, polygenic resistance is also inevitably more non-specific than major-gene resistance [25], [27], with potential to affect a broad spectrum of invading genotypes. Thus, it is conceivable that efforts to breed polygenic DED resistance into elms could have unintentional effects on the endophytic communities.

In the presented study, we hypothesized that elms with a high tolerance to DED host a less rich endophytic community than highly susceptible elms, due to their stronger defences, which are conferred by multiple genes. It should be noted that this hypothesis does not exclude the possibility that the phenotypic resistance shown by a given elm tree might be conferred by specific endophytes [28]–[30], either via direct antagonistic effects on pathogens or indirectly via the induction of specific plant responses, such as the production and release of defensive metabolites. Our study system allowed us to compare the constitutive phenolic profiles of elm genotypes with different degrees of DED resistance, and to evaluate the importance of tissue-specific phenolic status with respect to both pathogens and endophytes. The significance of the results for tree breeding and biodiversity conservation is discussed.

## Methods

### Ethics Statement

All necessary permits were obtained for the described field studies. One of the study sites, the Rivas-Vaciamadrid site is privately-owned. Oral permission for collection of samples was obtained from the landowner, Ms. Ana María Hernández Ros. For the activities at the Forest Tree Breeding Centre no specific permission was required. The Centre is governmentally-owned and the studies were conducted as part of the regular research activities under supervision of the Head of the Center, Mr. Salustiano Iglesias. The studies did not involve endangered or protected species.

### Sites and Plant Material

**Figure 1:**
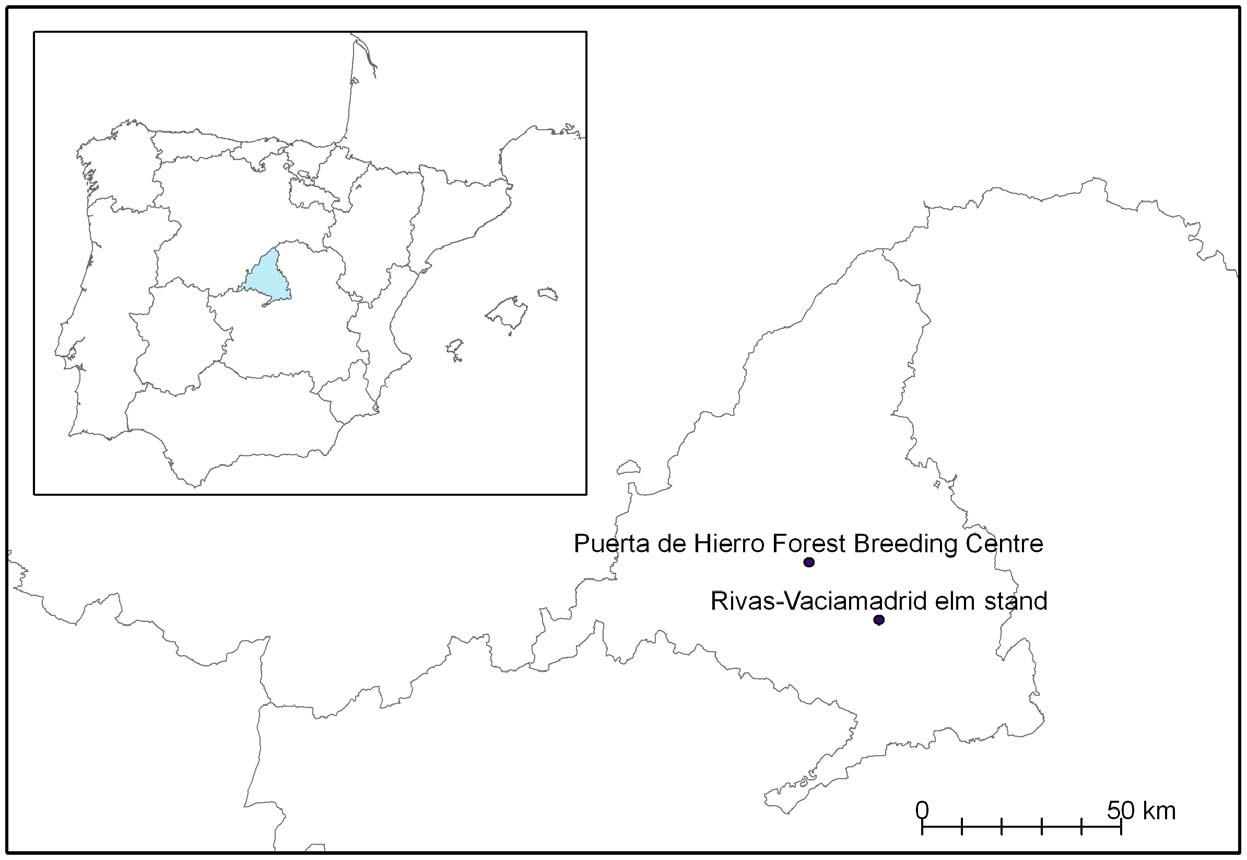
Location of the two study areas in central Spain.

Elms from two sites in the vicinity of Madrid, Spain were studied (Fig. 1). The first site is located at the Forest Breeding Centre in Puerta de Hierro (40°27′N, 3°46′W), and comprises 205 elm clones randomly planted with a spacing of 4×4 m in a conservation plot (152×36 m) with uniform microclimatic conditions. Each clone was represented by a single 14-year-old ramet. The distance between the selected clones ranged from 16 to 100 m, without any spatial grouping among resistant and susceptible clones. For our study, four *U. minor* and two *U. pumila* clones with low susceptibility to DED were selected (hereafter referred to as *resistant* clones), along with four *U. minor* clones that are highly susceptible to DED (*susceptible* clones; Table 1). The number of clones selected for study was determined by the availability of resistant trees (*U. minor* and *U. pumila*) of the same age and information of their different genetic background [31], [32]. The soil has a sandy loam texture and was amended annually with organic matter to enhance moisture retention. The plot was irrigated by sprinklers during spring and summer to avoid water stress.

**Table 1:** Specifications of the plant material growing at *Puerta de Hierro* Forest Breeding Centre, Madrid, Spain [P (R) = resistant *U. pumila* clones; M (R) = resistant *U. minor* clones; M (S) = susceptible *U. minor* clones].

Species	Tree group	Code	Origin	Susceptibility to DED(% wilting^a^)
***U. pumila*** b	P (R)	201	Nanyiang, Henan, China	low (7±14)
		203	Shangqiu, Henan, China	low (24±18)
*U. minor*	M (R)	UPM007^c^	Alatoz, Albacete, Spain	low (27±10)
		UPM072	Cazorla, Jaén, Spain	low (31±12)
		UPM093	Dehesa de la Villa, Madrid, Spain	low (25±12)
		UPM130	Pedrizas, Málaga, Spain	low (28±11)
	M (S)	UPM045	Ruidera, Ciudad Real, Spain	high (94±13)
		UPM068	Huélago, Granada, Spain	high (90±15)
		UPM158	San Nicolás, Sevilla, Spain	high (80±18)
		UPM171^d^	Puebla de Montalbán, Toledo, Spain	high (91±8)

a Values obtained from a previous susceptibility test [84].

b Provided by the Institute of Forestry and Nature Research (Wageningen, The Netherlands).

c Morphologically appears to be *U. minor* × *U. pumila.*

d *U. minor* var. *vulgaris* ( =  *U. procera*).

The second study site is a semi-natural riparian elm stand located in the municipality of Rivas-Vaciamadrid (40°20′N, 3°33′W) consisting of about 270 *U. minor* trees, all of which are between 65 and 75 years old [33]. It is the best-conserved elm stand in Madrid where *U. minor* is the dominant tree species. With a distance of 30 km it is also the closest stand to the Breeding Centre with *U. minor* as the dominant tree species. Most of the trees in the Rivas-Vaciamadrid stand belong to the unique, highly susceptible *U. minor* var. *vulgaris* clone [32]. This taxon presents very low genetic variability, probably because it originates from a single *U. minor* tree, the Atinian elm [32]. Thus, these trees are genetically close to the *U. minor* var. *vulgaris* clone UPM171 at the Breeding Centre. Since 2001, *O. ulmi* and *O. novo-ulmi* have been isolated from several trees of the stand [34], and *Scolytus* bark beetles are abundant in the area [33]. Despite these factors, the spread of DED in the stand is slow, suggesting environmental control of the disease. The Rivas-Vaciamadrid elm stand has historically been used as cattle raising area, where disinfectant products based on phenolic compounds (mainly phenol) were repeatedly applied to the cattle or to the soil to prevent insect bites and hoof infections. The same compounds have been shown to have a strong antifungal activity against *O. novo-ulmi* and induce the accumulation of suberin-like compounds in xylem tissues [35]–[37]. Seven *U. minor* var. *vulgaris* trees were selected from the stand on the basis of their similar dendrometric features (20.00±3.11 m in height; mean ± SD), good health condition and availability of information about their taxonomy [33].

The four tree groups were coded as follows: **P (R)**, resistant *U. pumila* clones from the Breeding Centre; **M (R)**, resistant *U. minor* clones from the Breeding Centre; **M (S)**, susceptible *U. minor* clones from the Breeding Centre; and **M (F)**, *U. minor* trees from the Rivas-Vaciamadrid field population.

### Sampling of Leaves and Twigs, and Isolation and Characterization of Endophytes

In mid May 2008, one terminal shoot (30 cm long) was cut from the lower half of the crown (at a height of 2 - 3 m) from each of the four cardinal points of the compass (i.e. four shoots from each elm tree). Four leaves were detached from each shoot in order to isolate endophytes (16 leaves per tree). Four 2-year-old twig segments (4 cm in length) were also cut from each shoot (16 twigs per tree) in order to isolate endophytes from bark and xylem tissues. Samples were transported in glass vials to the laboratory.

The leaves were surface-disinfected by dipping in 75% ethanol (30 s), 4% Na-hypochlorite (1 min) and 75% ethanol (15 s) [38]. After air drying (4 min), a disc with a diameter of 10-mm was cut aseptically from a randomly selected region of each leaf and placed on 2% (w/v) malt extract agar with no added antibiotics in Petri dishes. Twig segments (8–10 mm in diameter) were surface-disinfected following the same procedure as used for leaves save that they were immersed in the Na-hypochlorite solution for 5 min rather than one. After air drying (8 min), one 4×4×10 mm (thickness, width, length) slice (including bark and xylem tissues) was cut aseptically from each twig segment. The bark (about 2 mm thick) was separated from the xylem, and both tissues were placed in separate Petri dishes containing 2% (w/v) malt extract agar with no added antibiotics. The sizes of the leaf, xylem and bark samples were selected so as to ensure that each tissue sample had a similar weight (30–40 mg). The Petri dishes were sealed with Parafilm. The isolation method used resulted in the growth of endophyte colonies which were counted and sub-cultured 2 weeks after incubation at 20°C. The efficacy of the sterilization method was previously tested by direct comparison of the rate and number of fungal colonies that grew from sterilized and unsterilized tissue samples. The results from these tests indicate that in over 90% of the cases, rapidly-growing epiphytic fungi could be removed by the sterilization process and that the recovered isolates thus represent mainly the tissue internal fungal communities.

The endophytes were grouped into morphotaxa on the basis of vegetative features that conservatively reconstruct species boundaries [29], [39]. In each tree group [P (R), M (R), M (S) or M (F)], *endophyte frequency* was calculated as the average of the number of endophytes colonies growing in each Petri dish divided by the total number of tissue samples placed in the dish (four samples per dish; i.e. four samples per tissue and cardinal point). *Endophyte diversity* of each tree group was estimated as the average of the number of different morphotaxa observed in each Petri dish divided by the number of tissue samples placed in the dish [38]. To describe and compare the fungal communities in different sample groups, we used diversity indices [40]. First, to compare the *diversity*, we calculated the Shannon-Weaver index [H′ = – sum (P_i_ ln[P_i_]) where P is the proportion of taxon i] and used it to calculate Pielou’s index for *evenness* [J′ =  H′/H′_max_, where H′_max_ = log(S) and S = number of taxa]. Higher values of H′ indicate higher diversity and less competition between the taxa, and higher values of J′ indicate low variation in the distribution of taxa across the community. Endophytic communities were also compared among tree tissues, genotypes and sites using the classical Jaccard’s *similarity* index, based on binary information (presence/absence), as described by Anderson et al. [41]. This index allows us to quantify the degree of overlap between the taxa in the two communities. The Jaccard’s index (J) was calculated as J = A/(A+B+C) where A =  number of taxa common to both communities; B =  the number of taxa present in community 1 but not 2; C =  the number of taxa present in community 2 but not 1. Higher values indicate higher similarity between the two communities.

### Identification of Endophytic Fungi

Macro- and microscopic examination of morphological traits was used to tentatively assign isolates to morphotaxa. In addition, the molecular identity of one representative isolate per morphotaxon was determined as described below, for more precise information on the identity of the fungal isolates. The criterion used when selecting isolates was that they had to clearly exhibit the vegetative traits of the morphotaxon they exemplified.

For isolation of DNA, the fungal isolates were incubated on 2% malt extract liquid medium (20 g l^−1^ malt extract) at 25°C for 4–7 days. The hyphal mass was centrifuged down (10060 g, 2 min). After washing with water, 200 µl of the lysis buffer (2% Triton X-100, 1% SDS, 0.1 M NaCl, 0.001 M EDTA, 0.01 M pH 8 Tris buffer ), 200 µl of a phenol:chloroform:isoamyl alcohol mixture (25∶24:1) and 100 µl of acid-washed glass beads were added to the fungal pellet. The resulting mixture was vortexed for 10 min and 200 µl of pH 8 TE buffer (10 mM pH 7.5–8 Tris, 1 mM pH 8 EDTA) was added. The suspension was centrifuged for 10 min at 10060 g and then 10 µl RNase A (10 mg ml^−1^) was added to the aqueous phase, which was incubated for 45 min at 37°C. The DNA was precipitated with 1 ml ice cold 96% ethanol and 3 M sodium acetate (1/10 volume). The mixture was centrifuged for 10 min at 10060 g at 4°C. The pellet was washed with ice cold 70% ethanol, air-dried and resuspended in 40 µl TE buffer (pH 8).

The internally transcribed spacer (ITS) region of the rDNA and the small ribosomal subunit (SSU) were amplified using the ITS1/ITS4 and NS5/NS6 primer pairs, respectively [42]. The polymerase chain reaction was run under the following conditions: 94°C, 5 min followed by 30 cycles of 95°C for 30 sec, 50°C for 45 sec and 72°C for 45 sec followed by a final ten minute extension step at 72°C. The PCR products were purified using the GeneJET PCR Purification kit (Fermentas, cat. no K0702) and sequenced using PCR primers by MWG Operon (Ebersberg, Germany). The sequences were identified by comparison with GenBank database using nucleotide megablast search (Table 2, Table S1) [43].

**Table 2:** Identification of representative isolates of the morphotaxa (1–16) on basis of the top three BLAST hits (based on nucleotide megablast of ITS rDNA sequences) with corresponding GenBank taxa identity, characteristic morphological colony traits and literature.

Morphotaxon	Suggested taxon
1	*Pyrenochaeta cava* (syn. *Phoma cava*) [85], [86]
2	*Monographella nivalis* (syn. *Fusarium nivale*, *Gerlachia nivalis, Microdochium nivale*) [75], [87]
3	*Aureobasidium pullulans* [78], [88]
4	*Alternaria* sp. *(A. tenuissima)*
5	*Cochliobolus cynodontis (*anam. *Bipolaris cynodontis)*
6	*Fusarium* sp.
7	*Alternaria alternata*
8	*Biscogniauxia nummularia (*syn. *Hypoxylon nummularium)*
9	*Xylaria* sp.
10	*Cladosporium cladosporioides*
11	*Phomopsis* sp.
12	*Sordaria fimicola*
13	*Coniochaeta* sp. *(*anam. *Lecythophora)*
14	*Apiospora* sp. *(*anam. *Arthrinium)*
15	*Botryosphaeria sarmentorum*
16	*Leptosphaeria coniothyrium*

### Chemical Analyses of Leaf, Bark, and Xylem Tissues

Additional leaf and twig samples were collected following the same procedure as described for the isolation of endophytes. The bark was separated from xylem using a knife and the samples were allowed to dry in paper bags at room temperature. The samples were then milled into a homogenous powder and 10 mg (leaves and bark) or 300 mg (wood) per tree were extracted with methanol an analysed by HPLC [44]. The peak area data was collected at 320 nm. The quantitative data is expressed as peak area (AU×10^−5^) normalized against sample weight per injection. In order to explore the type of phenolic compounds present in the samples, UV-absorbance scanned at 200 to 400 nm was compared to spectral data in an in-house standard compound library. A more comprehensive identification of all compounds was not deemed crucial to fulfil the objectives of this study because we were mainly interested in screening the general patterns among the studied trees, their resistance and endophyte status.

### Statistical Analyses

Endophyte frequency and diversity were analyzed using a generalized linear model (GLM) approach to ANOVA with type III sum of squares, considering the effects of the *group* P (R), (M (R), M (S), and M (F), the *tree* nested within the *group*, the *organ* (leaf, bark, and xylem), the *orientation* (North, South, East and West), and the two-fold interactions between organ and orientation. The normality of the data was confirmed using the Shapiro-Wilks statistic [45]. The mean frequency and diversity values were compared by means of multiple range tests using Fisher’s least significant difference (LSD) intervals (α = 0.05). Linear regression analyses were made between the susceptibility to DED of each elm clone at the Breeding Centre (% leaf wilting) and the frequency and diversity of endophytes in xylem tissues. A non-metric multidimensional scaling (MDS) analysis based on the Jaccard similarity index matrix of any given pair of samples was performed to visualize any grouping in the data set.

To compare morphotaxa richness in tree groups with different sample sizes, and to summarize the completeness of the sampling effort, sample-based rarefaction curves [46] (hereafter referred to as endophyte accumulation curves) of the endophyte morphotaxa (abundance data) were constructed with EstimateS 8.2.0 software using 100 randomizations, sampling without replacement and default settings for upper incidence limit for infrequent species [47].

In order to compare the phenolic profiles of leaf, bark, and xylem samples from each tree group, the results obtained from HPLC analysis were tested using a discriminant function analysis (DFA). The chemical profile of each sample was defined on basis of a characteristic pattern of chromatogram peaks (13 peaks for leaf and bark samples, and 10 peaks for xylem samples), whose normalized areas were used as input variables with a priori information about sample grouping in the data (tree groups). This information was used to produce measures of within-group variance and between-group variance and then to define optimised discriminant functions (DFs) for distinguishing between profiles originating from different groups of trees. In order to estimate the discriminating power of the DFs, Wilks’ Lambda tests were performed. The coefficients by which the original variables (peak retention times) are multiplied to obtain the DFs are called loadings. Since the numerical value of a loading of a given variable on a DF indicates how much the variable has in common with that DF, loadings were used to identify the peaks that were most important in discriminating between samples. The areas of these significant peaks were compared within groups of trees by means of one-way ANOVA. Fisher’s least significant difference (LSD) procedure was used to compare averages (*α = *0.05). All statistical analyses were performed using Statistica 7.0 software package (Tulsa, OK, USA).

## Results

### Endophyte Frequency and Diversity

The ANOVA of the endophyte *frequency* revealed that the tree group [P (R), M (R), M (S) and M (F)], the tree nested within the group, and the organ all had significant effects on endophyte frequency (*P*<0.04), but the orientation and the organ-orientation interaction did not (*P*>0.70). Considering all tree groups, the endophyte frequency in bark tissues (0.66±0.03; mean ± SE) was higher (*P*<0.001) than in leaves (0.19±0.04) and xylem tissues (0.10±0.03). The ANOVA of the endophyte *diversity* showed that the tree group, the tree nested within the group, and the organ had significant effects on endophyte diversity (*P*<0.01), but the orientation and the organ-orientation interaction did not (*P*>0.77). Considering all tree groups, the endophyte diversity in bark tissues (0.47±0.03) was higher (*P*<0.001) than in leaves (0.14±0.03) and in xylem tissues (0.07±0.02).

The total number of fungal isolations obtained from the different plant tissues and tree groups is specified in Table 3. A total of 274 isolations were obtained from the 816 plant samples incubated on MEA. The endophytic fungi were classified into 16 different morphotaxa. Six of these were isolated exclusively from bark, three from bark and leaves, and three from bark and xylem; the remaining four morphotaxa were isolated from all tissue types. According to the Shannon-Weaver index (H′, Table 3), the leaf-associated isolates showed a markedly higher diversity and evenness in M (F) trees, as compared to those from the Breeding Centre. For bark tissues, the differences in H′ and J′ values among the tree groups were not as pronounced as they were for leaf or xylem tissues, and the highest diversity and evenness were found for the M (S) group (Table 3). Also for xylem tissues, the H′ and J′ indices suggest highest diversity and evenness, and thus lowest competition, in the M (S) group (Table 3).

**Table 3:** Number of tissue samples (incubated on MEA at 20°C), fungal isolates and morphotaxa obtained, and associated diversity indices: H′ = Shannon-Weaver and J′ = Pielou’s evenness index [tree groups: P (R) = resistant *U. pumila* clones from Puerta de Hierro Forest Breeding Centre; M (R) = resistant *U. minor* clones from Puerta de Hierro Forest Breeding Centre; M (S) = susceptible *U. minor* clones from Puerta de Hierro Forest Breeding Centre; and M (F) = *U. minor* trees from Rivas-Vaciamadrid field site].

Organ	Indices	P (R)	M (R)	M (S)	M (F)
Leaf	Number of tissue samples	32	64	64	112
	Number of isolates	6	4	6	50
	Number of morphotaxa	3	2	2	5
	H′	0.56	0.26	0.34	1.44
	J′	0.51	0.37	0.49	0.89
Bark	Number of tissue samples	32	64	64	112
	Number of isolates	19	42	45	76
	Number of morphotaxa	8	10	13	9
	H′	1.65	1.96	2.31	1.8
	J′	0.79	0.85	0.90	0.82
Xylem	Number of tissue samples	32	64	64	112
	Number of isolates	1	2	18	5
	Number of morphotaxa	1	2	7	2
	H′	0.15	0.17	0.94	0.22
	J′	0	0.24	0.48	0.32
All tissues	Number of tissue samples	96	192	192	336
	Number of isolates	26	48	69	131
	Number of morphotaxa	8	11	14	11
	H′	1.81	1.95	2.28	2.18
	J′	0.87	0.81	0.86	0.91

**Figure 2:**
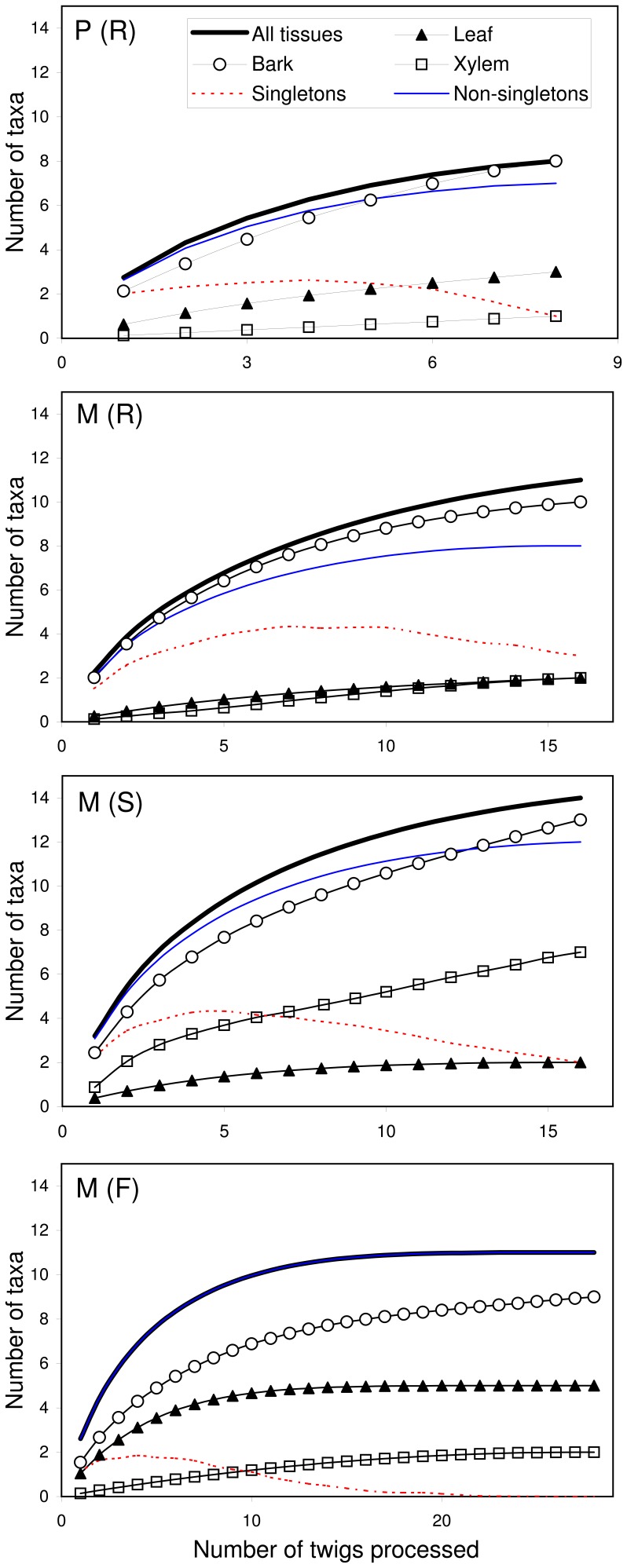
Accumulation curves of elm endophytic fungi. Accumulation curves indicating the number of endophyte morphotaxa isolated per number of twigs processed (four twigs per tree, and four leaf, bark and xylem samples per twig) in each tree group [P (R) = resistant *U. pumila* clones from Puerta de Hierro Forest Breeding Centre; M (R) = resistant *U. minor* clones from Puerta de Hierro Forest Breeding Centre; M (S) = susceptible *U. minor* clones from Puerta de Hierro Forest Breeding Centre; and M (F) = *U. minor* trees from Rivas-Vaciamadrid field site].

The sample-based rarefaction curves constructed for individual tissues showed different patterns: within each tree group, the curves for bark tissue increased at highest rate and reached the highest end points, whereas the curves constructed for xylem and leaf samples increased slower and remained at lower levels throughout the empirical range of samples (Fig. 2). Within this range, the curves constructed for bark tissues approached asymptote in all tree groups, and those for the xylem and leaves clearly reached a plateau in M (F) group. The highest end points of the curves constructed for bark and xylem, as well as for all tissues, were found in M (S) group (Fig. 2). The sample-based rarefaction curves based on non-singletons of all tissues reached an asymptote in all tree groups (Fig. 2). After an initial increment, the number of singletons diminished progressively as the number of twigs processed increased (Fig. 2). The initial level of singletons was lowest in M (F) group, reaching zero when the number of processed twigs was 26.

**Figure 3:**
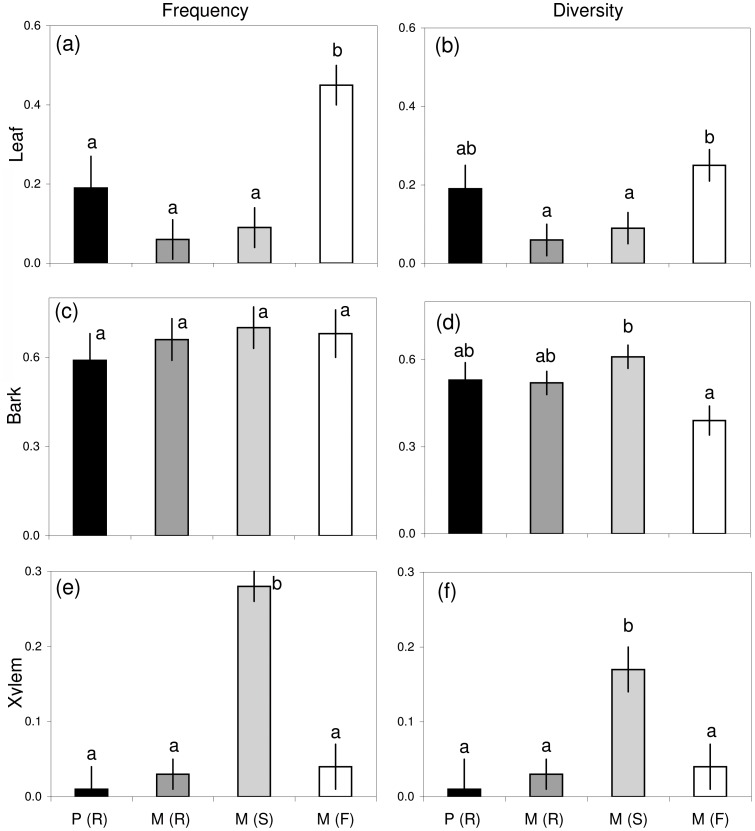
Endophyte frequency and diversity in elms. Mean values of endophyte frequency (a, c, e) and endophyte diversity (b, d, f) of leaf (a, b), bark (c, d), and xylem (e, f) tissues from different groups of elm trees: P (R) = resistant *U. pumila* clones from Puerta de Hierro Forest Breeding Centre; M (R) = resistant *U. minor* clones from Puerta de Hierro Forest Breeding Centre; M (S) = susceptible *U. minor* clones from Puerta de Hierro Forest Breeding Centre; and M (F) = *U. minor* trees from Rivas-Vaciamadrid field site. Different letters indicate differences among groups of trees (*P*<0.05), and bars represent standard errors.

The endophyte frequency and diversity for each group of trees and tree organs were compared on basis of mean values and multiple range test comparisons (Fig. 3). In leaf tissues, the M (F) group showed a higher endophyte frequency than the other groups (*P*<0.05; Fig. 3a), and a higher endophyte diversity than the M (R) and M (S) groups (*P*<0.05; Fig. 3b). In bark tissues, no significant differences in endophyte frequency were observed between the groups (*P*>0.12; Fig. 3c), while M (S) showed higher diversity than the field population (*P*<0.05; Fig. 3d). In xylem tissues, both frequency and diversity values were higher in M (S) than in the rest of tree groups (*P*<0.05; Fig. 3e, f).

**Figure 4:**
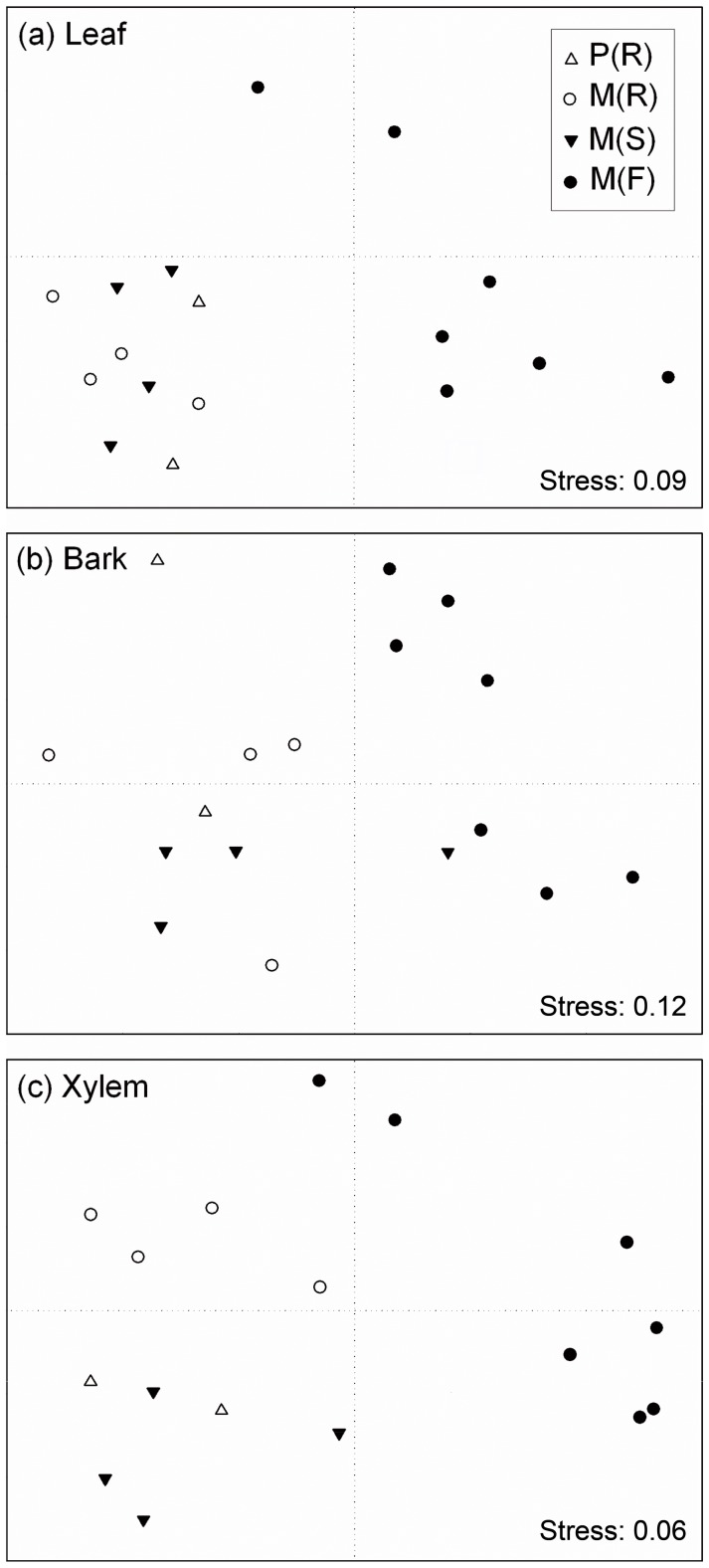
Two-dimensional ordination using non-metric multidimensional scaling (MDS) based on Jaccard’s similarity measures. Each point represents the fungal endophyte community of an individual tree. Endophytes were isolated from leaf (a), bark (b) or xylem (c) tissues. Groups of elm trees: P (R) = resistant *U. pumila* clones from Puerta de Hierro Forest Breeding Centre; M (R) = resistant *U. minor* clones from Puerta de Hierro Forest Breeding Centre; M (S) = susceptible *U. minor* clones from Puerta de Hierro Forest Breeding Centre; and M (F) = *U. minor* trees from Rivas-Vaciamadrid field site.

The MSD graph obtained from the Jaccard’s similarity matrix showed a clear distinction in leaf endophyte community between the M (F) trees and the trees from the Breeding Centre (Fig. 4a). The same analysis applied to the bark endophytes revealed a higher overlap among tree groups than in leaf or xylem tissues (Fig. 4b). However, M (F) samples were grouped in the positive horizontal semi-axis together with a M (S) tree from the Breeding Centre. This M (S) tree is the UPM007 clone (Table 1), belonging to the *U. minor* var. *vulgaris* complex, which also includes the trees studied at the field population. For the xylem-associated endophyte communities (Fig. 4c), a clear distinction was again observed between M (F) and the trees from the Breeding Centre. Furthermore, a clear distinction in endophyte diversity was observed between the M (R) trees on the one hand, and the M (S) and P (R) trees on the other hand (Fig. 4c).

Morphotaxa 3, 4, and 8 were isolated from all tree groups from the Breeding Centre, but not from the field population. Morphotaxon 13 was only isolated from one resistant *U. minor* clone (UPM007) and from one resistant *U. pumila* clone (201). Morphotaxon 7 was exclusive to *U. minor* var. *vulgaris*, since it was only isolated from the UPM171 clone and trees from the field site. Morphotaxon 14 was exclusively isolated from the resistant *U. minor* clone UPM007, while morphotaxa 15 and 16 were only isolated from the susceptible *U. minor* clones UPM045 and UPM068. It is noteworthy that five endophytic morphotaxa (3, 4, 6, 10 and 15) were isolated from the xylem of susceptible *U. minor* clones from the Breeding Centre, but not from the xylem of other tree groups (data not shown). However, four of these morphotaxa (3, 4, 6 and 10) were not restricted to susceptible *U. minor* clones, as they were also isolated from leaf or bark tissues from other tree groups.

The three most common fungal morphotaxa were characterized by *Pyrenochaeta cava* (morphotaxon 1), *Monographella nivalis* (morphotaxon 2), and *Aureobasidium pullulans* (morphotaxon 3) (Table 2, Table S1). Of these, *M. nivalis*, isolated mainly from bark and occasionally from xylem, was predominantly associated with resistant *U. minor* clones and *U. minor* trees from the field population, *P. cava* was primarily associated with the resistant *U. pumila,* and *A. pullulans* was primarily associated with susceptible *U. minor* clones. According to the molecular analysis, the other morphotaxa included species from the genera *Alternaria* (morphotaxa 4 and 7), *Bipolaris* (morphotaxon 5), *Fusarium* (morphotaxon 6), *Biscogniauxia* (morphotaxon 8), *Xylaria* (morphotaxon 9), *Cladosporium* (morphotaxon 10), *Phomopsis* (morphotaxon 11), *Sordaria* (morphotaxon 12), *Coniochaeta* (morphotaxon 13), *Apiospora* (morphotaxon 14), *Botryosphaeria* (morphotaxon 15), and *Leptosphaeria* (morphotaxon 16) (Table 2, Table S1). The Dutch elm disease pathogen was not isolated from any sampled tree in this study.

**Figure 5:**
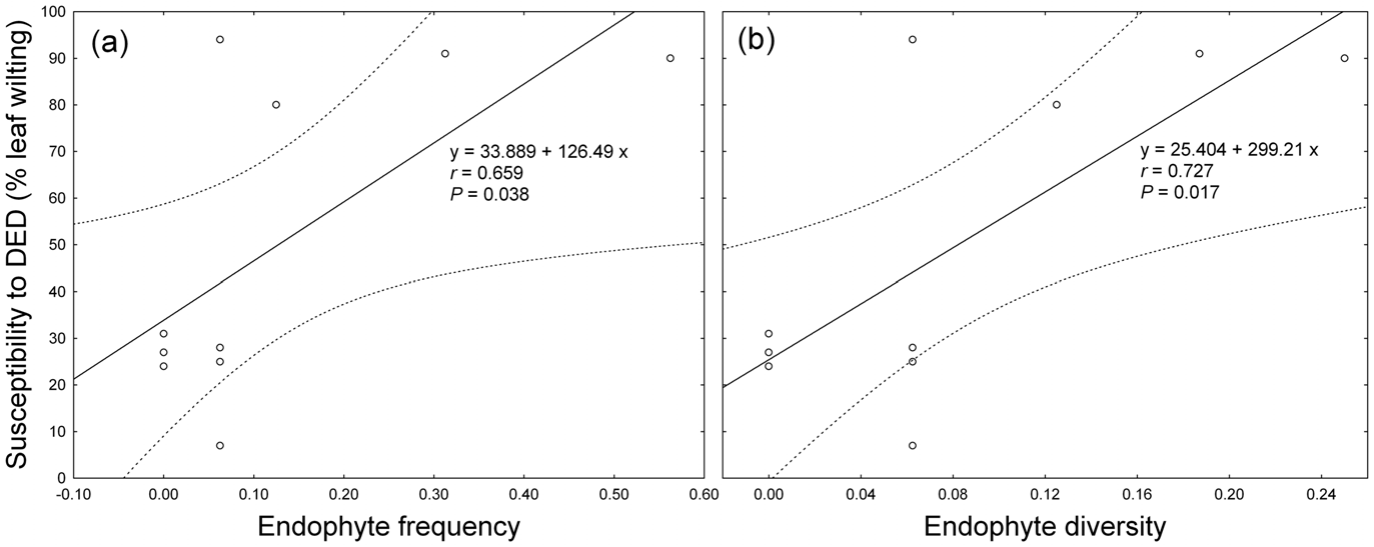
Relation between endophytes and susceptibility to DED in elms. Relations between the mean susceptibility to DED (% leaf wilting) of each elm genotype at the Breeding Centre and its endophyte frequency (a) and diversity (b) in xylem tissues. Solid lines are linear regressions and dotted lines are 95% confidence limits. Wilting values were obtained from a previous susceptibility test [84].

In xylem tissues, the endophyte frequency and diversity of each genotype at the Breeding Centre were directly related with their mean susceptibility to DED (Fig. 5) (*r* = 0.659, *P* = 0.038; *r* = 0.727, *P* = 0.017, respectively).

### Chemical Discrimination of Leaf, Bark, and Xylem Tissues

Quantitative and qualitative differences between the different tissues’ phenolic profiles were identified. Several phenolic acids (coumaric acids and chlorogenic acids) were tentatively identified in the leaf samples, along with flavonoids (quercetin and kaempherol derivatives). Bark tissues contained several compounds whose UV-spectra resembled those of catechins and eriodictyol, along with quercetin and kaempherol-type flavonoids, albeit at lower concentrations than were observed in the leaves. The phenolic acid pool in the xylem samples was rich in compounds identified as rosmarinic acid, vanillic acid and chlorogenic acid.

**Figure 6:**
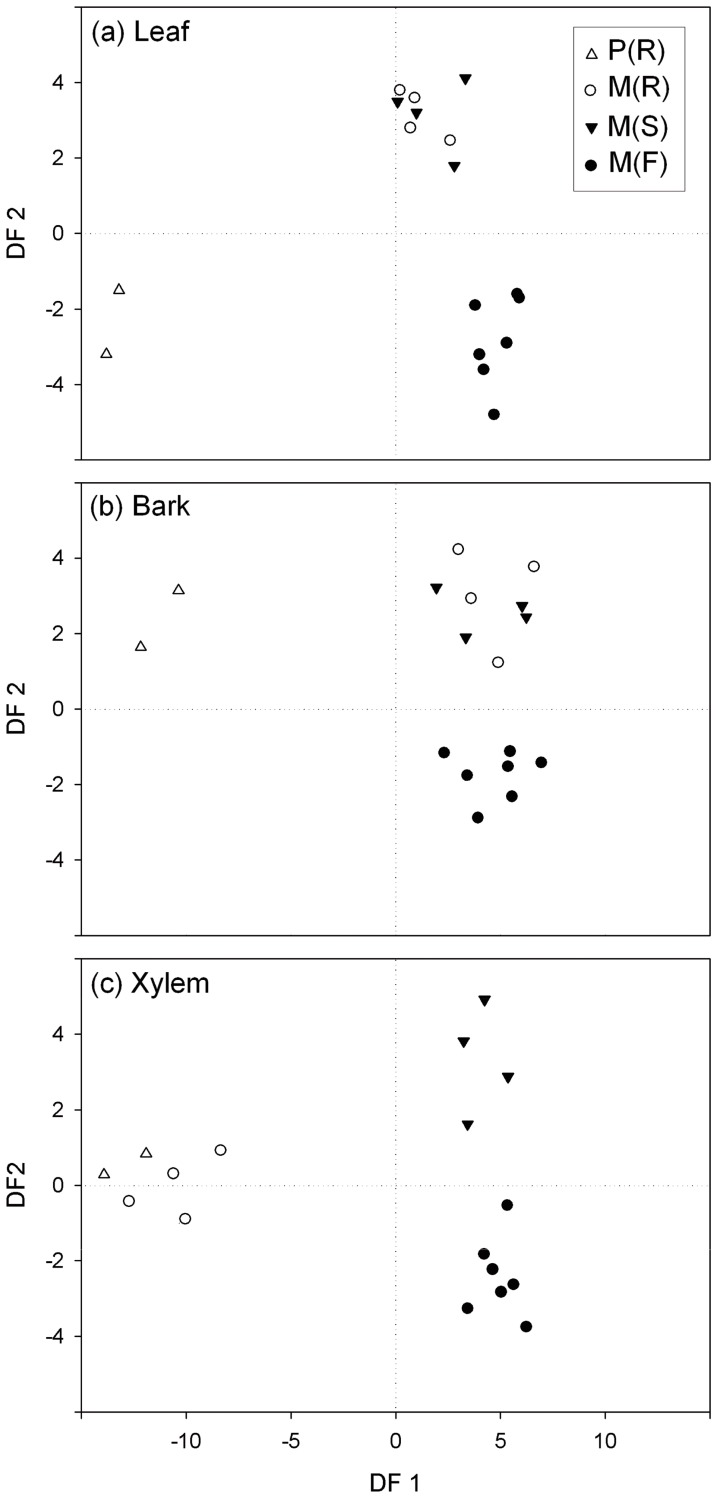
Separation of elm trees on basis of tissue specific phenolic profiles. Discriminant function analysis score scatter plot for the HPLC chromatogram peaks of samples taken from leaf (a), bark (b), and xylem (c) tissues from different groups of trees: P (R) = resistant *U. pumila* clones from Puerta de Hierro Forest Breeding Centre; M (R) = resistant *U. minor* clones from Puerta de Hierro Forest Breeding Centre; M (S) = susceptible *U. minor* clones from Puerta de Hierro Forest Breeding Centre; and M (F) = *U. minor* trees from the Rivas-Vaciamadrid site.

The DFA of the chemical variables (chromatogram peaks) was used to obtain the scatter plot of the scores from the first two DFs (Fig. 6). For the leaf samples (Fig. 6a), DF1 was significant at *P*<0.001, and could be used to distinguish between *U. minor* (positive scores) and *U. pumila* (negative scores) samples (Fig. 6a). DF2 (*P* = 0.02) could be used to distinguish between *U. minor* samples from the Breeding Centre [both M (R) and M (S)] and those from the field site [M (F)].

A similar discrimination pattern was observed with bark tissues (Fig. 6b): DF1 (*P*<0.001) could be used to distinguish between *U. minor* (positive scores) and *U. pumila* (negative scores) samples, while DF2 (*P* = 0.01) separated the *U. minor* samples from the Breeding Centre [both M (R) and M (S)] and those from the field site [M (F)].

For the xylem samples (Fig. 6c), DF1 (*P*<0.001) could be used to distinguish between M (S) and M (F) samples from P (R) and M (R) samples (negative scores), while the discriminating power of DF2 was not statistically significant (*P* = 0.15).

**Figure 7:**
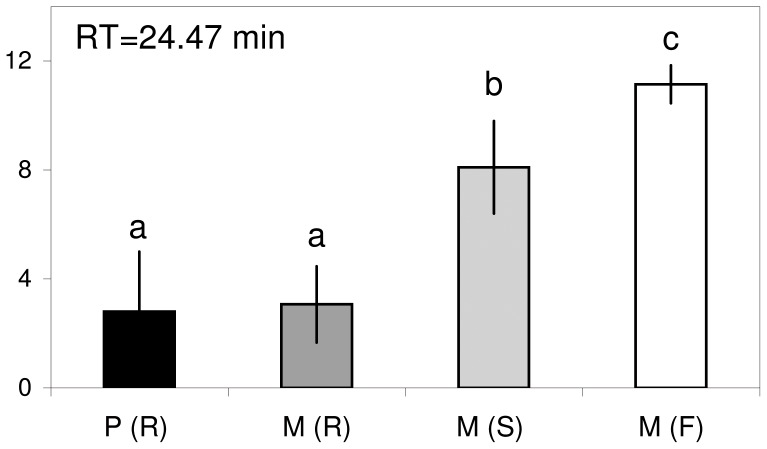
Quantitative patterns of a rosmarinic acid derivative in elms. Mean peak area (AU×10^−5^) of one of the HPLC chromatogram peaks (RT = 24.47 min) of xylem samples that was important in discriminating between tree groups: P (R) = resistant *U. pumila* clones from *Puerta de Hierro* Forest Breeding Centre; M (R) = resistant *U. minor* clones from *Puerta de Hierro* Forest Breeding Centre; M (S) = susceptible *U. minor* clones from Puerta de Hierro Forest Breeding Centre; and M (F) = *U. minor* trees from Rivas-Vaciamadrid field site. Different letters indicate differences between groups of trees (*P*<0.05); bars represent standard errors [n = 4 for M(R) and M(S), 2 for P(R) or 7 M(F)].

The chromatogram peak at 24.47 min, identified as rosmarinic acid derivative, was one of the most significant peaks in discriminating between xylem samples. The area of this peak showed higher mean values for genetically susceptible trees [M (S) and M (F)] than for resistant trees [M (R) and P (R)] (Fig. 7).

## Discussion

Our study shows that DED-susceptible *U. minor* clones may harbour a greater range and higher densities of endophytic fungi in their xylem tissues than resistant *U. minor* and *U. pumila* clones. Since the DED pathogen develops in xylem [16], the genetic features that increase the constitutive resistance of elms to *O. novo-ulmi* may also negatively affect endophytic fungi in their xylem, leading to a trade-off between fungal biodiversity and DED resistance. The highest end points of the accumulation curves for xylem in M (S) group also support the view that these trees, with high susceptibility to DED, sustain richer endophyte communities in their woody tissues than the more resistant trees. However, the higher fungal diversity and evenness, which indicates an environment with lower level of competition between different fungi, found in the xylem of susceptible trees (see the Shannon’s and Pielou’s indices in Table 3), should not be generalized to other tissues, as most fungal morphotypes isolated from the xylem of susceptible trees were also isolated from bark or leaf tissues of resistant trees. Furthermore, these results should not be generalized to the entire fungal community of the elm trees, as our study was restricted to endophytic fungi isolated in malt extract agar. This medium permits the growth of most species of fungi once they are obtained in a pure culture. However, the initial growth and isolation of some slow-growing fungi may have been inhibited by the rapidly growing fungi. To achieve the isolation of the total culturable community, isolation conditions should permit equal expression of the entire array of fungal groups present; e.g. by restriction of the rapidly growing fungi by means of destructive chemical and physical procedures to support slow-growing fungi [48]. However, despite the limitations of our isolation protocol, we were able to find differences between susceptibility groups. The endophyte accumulation curves suggest that the sampling effort of 16 processed twigs from four elm trees (64 tissue samples) captured well the majority of the culturable endophytes. However, a more exhaustive sampling of about 20 twigs (i.e. 5 trees in the sampling design) can improve the catchment of the more rare or transient morphotypes. For extensive comparisons of the total fungal communities, pyrosequencing could be applied in future studies [49], [50].

Our results emphasize the strong effect of tree genotype on endophyte communities. It is noteworthy that the UPM007 clone from the Breeding Centre appeared in the MDS graph of bark tissues (Fig. 4b) close to the M (F) trees. All these trees belong to the *U. minor* var. *vulgaris* complex and therefore are genetically close to each other. This finding underlines the importance of maintaining the genetic diversity in tree populations. The significance of genetic variation of trees as a factor shaping the fungal assemblages has also been shown in the phyllosphere of European beech (*Fagus sylvatica*) [51]. Moreover, although the benefit of restoring elm stands through resistance breeding is obvious, the putatively high importance of endophytic fungi in forest ecosystems warrants careful consideration of the effect of resistance breeding. Previously, non-targeted effects of improved resistance have been studied mainly in transgenic plants. Newhouse et al. [26] found no negative effects of transformation with a gene encoding a synthetic antimicrobial peptide on mycorrhizal colonization in young elms (*U. americana* L.). Similar results have also been found in some other studies of plant-pathogen systems [52], but others have found that transgenic resistance may be accompanied by unintentional alterations in mutualistic fungal community [53]. Thus, results of increasing resistance transgenically have been mixed in this respect. However, compared to genetic modifications that only involve a limited number of genes, alterations in quantitative resistance traits may potentially cause more profound alterations in endophytic community.

In plant-endophyte interactions, immunity triggered by microbe-associated molecular patterns (MAMPs) does not ward off the interacting endophyte, as it remains hosted by the plant. Endophytes that have evolved closely with their host plants [54], [55] might produce MAMPs that activate signalling networks similar to those activated by beneficial microbes [56], resulting in only a mild induction of the plant’s immune responses [57]. Systemic resistance induced by these beneficial organisms appears to be predominantly based on priming for enhanced defence, rather than on direct activation of defence [57]. Further studies using in vitro model systems [58] are needed to clarify the biochemical interactions between trees and their endophytic fungi.

Anatomical features of the xylem may play a key role in elm resistance to DED [59], [60], but the variations in host anatomy alone cannot fully explain the variations in degrees of elm resistance to DED [60], [61]. A potentially contributory factor, although often neglected in studies on plant quality, is that endophytes may modify the chemical quality of plants [62]. We found that the phenolic profiles of xylem samples from resistant *U. minor* and *U. pumila* clones grouped together in the DFA, suggesting a link between xylem’s chemicals and DED. Further, in xylem tissues, some endophytic morphotaxa were exclusively found in susceptible *U. minor* genotypes, which also had high xylem concentrations of a compound identified as rosmarinic acid. It is possible that certain endophytes stimulate the accumulation of specific compounds in host tissues. For example, rosmarinic acid has been found to be induced by symbiotic mutualistic fungi (arbuscular mycorrhiza) in herbaceous plants [63]. However, other studies have provided evidence for a negative relation between polymeric phenolics (condensed tannins) and fungal endophyte infections in bark [64]. Obviously, the relation between fungal colonizers and phenolic end products can be multifaceted, because the phenolics could both affect, and be affected, by the fungi, and because structurally and functionally different phenolics might have different roles in host-endophyte interactions [21]. Moreover, some endophytes may be latent pathogens [5], [28] and be differently affected by the host chemicals at different physiological phases of their life-style continuum. A detailed identification of the compounds involved in the chemical discrimination of resistant and susceptible elm clones is in progress to further explore the relationships between these chemicals, endophytes and resistance in elms. While the host tree’s chemical quality may be an important factor affecting the endophytes, it should also be noted that the diversity of endophytes can also be strongly affected by several other factors, such as genotype or geographic differences. In addition to highlighting the potential importance of intrinsic factors in plant-endophyte interactions, our results underline the significance of environmental factors for endophyte diversity in trees. The frequency and diversity of the endophytic fungi (Fig. 3), and the Shannon’s and Pielous’s indices (Table 3) were rather similar in the xylem of the Rivas-Vaciamadrid elms, which are genetically susceptible but phenotypically resistant to *O. novo-ulmi*, and in the resistant genotypes growing at the Breeding Centre. This could be because the establishment of some xylem endophytes is hampered at Rivas-Vaciamadrid by the intensive application of phenolic cattle disinfectants that also prevent *O. novo-ulmi* spread there [33], [35]–[37]. However, the trees growing in the field at Rivas-Vaciamadrid had higher leaf endophyte frequencies, diversity and evenness (Fig. 3, Table 3), which may be explained by differences in the availability of fungal inocula. At the Breeding Centre, the soil is periodically ploughed and amended to enhance soil water retention and eliminate competition from herbaceous vegetation. This soil treatment buries plant materials, which probably reduces the availability of fungal inocula. Other environmental factors, such as the higher humidity associated with the riparian habitat of the Rivas Vaciamadrid elm stand may also favour a higher abundance of leaf endophytic fungi [65].

The differences found between the two study sites in terms of their fungal communities can be attributable to environmental factors, but also to differences in tree age or even to tree age × site interactions. It has been shown that plant age can affect the degree of plant colonization by endophytes. For instance, the infection density in leaves of woody plants tends to increase with leaf age [66]–[68]. In *Populus* × *euramericana*, endophyte richness in leaves and twigs was higher in young stands than in adult stands. Furthermore, the differences in richness between ages depended on the site quality [69]. In our study, it is not possible to ascertain how the tree age affected fungal communities, because all the trees within each location (Breeding Centre or Rivas-Vaciamadrid) were of approximately same age. However, it is possible that the strong “site” effect observed in fungal diversity (Fig. 4) was at least partly due to differences in plant age.

The faster increment of the accumulation curve for bark samples indicates greater richness or evenness of culturable endophyte morphotaxa in bark tissues, as compared to leaves and xylem. In all of the studied trees, also the frequency and diversity of endophytic fungi in the bark tissues was substantially greater than in the leaves, as has previously been reported [70]. This could be expected because bark tissues are colonized on a cumulative basis, with fungi persisting from year to year, whereas leaves are gradually colonized over the course of a single growing season. Conversely, xylem tissues are colonized more selectively [71].

The most abundant morphotypes were *P. cava, M. nivalis* and *A. pullulans*. Earlier, *P. cava* has been reported as an endophytic species involved in the aetiology of decline of Mediterranean *Quercus* trees [72], [73]. The fungus *M. nivalis* is a snow mold with a temperature minimum of −5°C for growth [74]. This fungus can cause severe damage on cereals and other grasses [75]. Its appearance as an endophyte in elm bark could be explained by the likely commonness of the species in the pasture lands surrounding the studied elms. The fungus *A. pullulans*, on the other hand, was an expected finding because it is a very abundant colonizer of plant surfaces and often isolated as an endophyte in trees [76], [77]. This polymorphic, yeast-like fungus is well-adapted to a broad range of habitats and is exploited for its ability to produce the biodegradable extracellular polysaccharide pullulan [78]. Similarly, several of the other tentatively identified genera, e.g. *Alternaria, Xylaria*, and *Phomopsis* have been reported as tree endophytes in earlier studies [29], [79]–[81].

The observed spatial variations in the diversity and frequency of fungal endophytes in elms, along with their associations with the elms’ chemical and resistance characteristics, emphasise the potential importance of endophytic fungi as dynamic modulators of tree phenotype. Nevertheless, it is difficult to assess whether the endophytic fungi are significant determinants of the phenotypic resistance observed at the Rivas-Vaciamadrid field site. We have recently found that *M. nivalis* (morphotaxon 2), predominantly associated with Rivas-Vaciamadrid trees and resistant *U. minor* clones, releases extracellular metabolites that in vitro inhibit *O. novo-ulmi* (K. Blumenstein et al., unpublished) and reduces the symptoms caused by *O. novo-ulmi* inoculation in elm trees previously challenged with the endophyte (Martín et al., unpublished). The presence of this endophyte could limit the spread of *O. novo-ulmi* in the inner bark of diseased trees, the compartment where the vector insects, elm bark beetles, become contaminated by spores. The potential of a bark endophyte (*Phomopsis oblonga*) to hamper the breeding of elm bark beetles has been previously reported [79], [80]. Our results from studies with *M. nivalis* indicate the existence of multiple mechanisms whereby endophytes can influence the DED transmission and the resistance of elms to *O. novo-ulmi* in field conditions.

In conclusion, we found support for our initial hypothesis: the resistant elm genotypes had a more limited endophytic flora in xylem tissues than the susceptible genotypes. However, a significant genotype effect was observed and not all susceptible genotypes showed higher values of endophyte frequency and diversity in xylem tissues than resistant genotypes (Fig. 5). Thus, it would be necessary to characterize the variation of the endophytic community of each genotype in greater detail, using 4–6 tree replicates per genotype. Currently, however, such elm material is not available in an adult stage, and to create it clonal propagation of the existing single genotypes would be necessary. Despite this reservation, our results imply that improving DED resistance in elm trees may have non-targeted effects on fungal biodiversity, and the re-introduction of elms to forest ecosystems with the assistance of breeding for quantitative resistance to DED may involve a trade-off between the goals of ecosystem restoration and fungal biodiversity conservation. As endophyte diversity may contribute to various ecosystem benefits from forests in a similar way than rhizospheric diversity, this issue should be addressed in environmental impact analyses of forest restoration and tree breeding efforts (see also [82], [83]). Obviously, the priority of elm breeding is to re-establish elm populations, and the re-introduction of resistant elms to the forests should increase potential habitats for endophytic fungi. Moreover, other plant species in the forest may act as reservoirs of cosmopolitan endophytes that inhabit also the susceptible elms. However, if the susceptible elm genotypes harbour specialist endophytes, a large-scale enrichment of resistant elm genotypes could impede their conservation. If these specialist endophytes are of particular relevance for wood degradation or ecological interactions, the ecosystem processes of the forest might change consequently. Future studies should thus explore further the diversity and ecological functions of the endophyte communities, including the non-culturable species, in elm genotypes differing in their resistance to DED.

## Supporting Information

Table S1 **The top three BLAST hits (based on nucleotide megablast of ITS rDNA sequences) with corresponding GenBank taxa identity and characteristic morphological colony traits of representative isolates for each morphotaxa (1–16) (“-“ = not determined).**
(DOCX)
