# Supplementary material for: Resistance to Dutch Elm Disease Reduces Presence of Xylem Endophytic Fungi in Elms (Ulmus spp.)
Source: PLoS One. 2013 Feb 28;8(2):e56987. doi: 10.1371/journal.pone.0056987 (PMC3585289; doi:10.1371/journal.pone.0056987)
Supplement: Table S1 — The top three BLAST hits (based on nucleotide megablast of ITS rDNA sequences) with corresponding GenBank taxa identity and characteristic morphological colony traits of representative isolates for each morphotaxa (1–16) (“-“ = not determined). (DOCX) [file pone.0056987.s001.docx]

**Table S1.** The top three BLAST hits (based on nucleotide megablast of ITS rDNA sequences) with corresponding GenBank taxa identity and characteristic morphological colony traits of representative isolates for each morphotaxa (1-16) (“-“ = not determined).

| **MT** | **NS** | | | **ITS** | | |  |
| --- | --- | --- | --- | --- | --- | --- | --- |
|  | **GenBank accession** | **Putative taxon** | **Coverage/**  **Identity** | **GenBank accession** | **Putative taxón** | **Coverage/**  **Identity** | **Macromorphology of the colony (growth rate on MEA, mm day^-1^)** |
| **1** | AY773746.1 | *Uncultured rhizosphere ascomycete* | 99/99 | AM901824.1 | Uncultured ascomycete | 100/97 | Colony grey-colored with greenish tone with fuzzy structure (1.5). |
|  | AY773806.1 | *Uncultured soil ascomycete* | 99/99 | AY354263.1 | *Pyrenochaeta cava* | 96/97 |  |
|  | AY857715.1 | *Lojkania enalia* | 98/99 | AY805638.1 | *Pyrenochaeta cava* | 94/96 |  |
| **2** | EF621487.1 | *Fusarium solani* | 98/99 | AB470850.1 | *Fusarium oxysporum* | 100/99 | Colony whitish to apricot/salmon or orange colored (2.4). |
|  | HQ219403.1 | *Uncultured dikarya* | 98/99 | AB369452.1 | *Fusarium tricinctum* | 100/99 |  |
|  | HQ219387.1 | *Uncultured dikarya* | 98/99 | AM901767.1 | Uncultured ascomycete | 100/99 |  |
| **3** | GU250318.1 | *Teratosphaeriaceae* sp. | 99/99 | FJ820754.1 | Uncultured fungus | 100/99 | Colony smooth, slimy, beige to pink colored, later with brownish or dark pigmented sectors (3.3). |
|  | JN546123.1 | *Aureobasidium pullulans* | 99/99 | EU755002.1 | *Dothioraceae* sp. | 100/99 |  |
|  | JF418148.1 | *Aureobasidium pullulans* | 99/99 | EU529999.1 | *Aureobasidium pullulans* | 100/99 |  |
| **4** | JQ390154.1 | *Alternaria* sp. | 99/99 | FJ755198.1 | *Alternaria tenuissima* | 98/98 | Colony center grey, furry; periphery greenish-brown and creamy (2.9). |
|  | JN088533.1 | *Alternaria alternata* | 99/99 | FJ755196.1 | *Alternaria tenuissima* | 98/98 |  |
|  | JF719098.1 | *Uncultured fungus* | 99/99 | FJ755194.1 | *Alternaria tenuissima* | 98/98 |  |
| **5** | JN941646.1 | *Cochliobolus cynodontis* | 99/98 | AY154691.1 | *Lewia infectoria* | 99/99 | Colony center grey, furry; periphery greenish-brown creamy (2.9). |
|  | JN941645.1 | *Cochliobolus cynodontis* | 98/98 | GU934538.1 | *Lewia infectoria* | 100/99 |  |
|  | AF229512.1 | *Alternaria petroselini* | 98/98 | FR822774.1 | *Alternaria sp.* | 98/99 |  |
| **6** | HQ219403.1 | *Uncultured dikarya* | 98/99 | AB470905.1 | *Fusarium* sp. | 100/100 | Colony white, with cottony structure (1.5). |
|  | HQ219387.1 | *Uncultured dikarya* | 98/99 | EF611091.1 | *Fusarium* sp*.* | 100/100 |  |
|  | EF621487.1 | *Fusarium solani* | 98/99 | JF740915.1 | *Fusarium* sp. | 100/99 |  |
| **7** | JQ390154.1 | *Alternaria* sp. | 96/99 | HQ385969.1 | *Alternaria compacta* | 99/100 | Colony white-grey, center furry, periphery creamy (2.8). |
|  | JN088533.1 | *Alternaria alternata* | 96/99 | JF835808.1 | *Alternaria alternata* | 99/99 |  |
|  | JF719098.1 | *Uncultured fungus* | 96/99 | JF835807.1 | *Alternaria alternata* | 99/99 |  |
| **8** | AF346563.1 | *Hypoxylon nummularium* | 99/88 | JF295128.1 | *Biscogniauxia mediterranea* | 96/99 | Colony white-yellow, with cottony structure; white/orange droplets, green spore accumulations (6.7). |
|  | AF346564.1 | *Hypoxylon mediterraneum* | 99/99 | JF295127.1 | *Biscogniauxia mediterranea* | 96/99 |  |
|  | - | *-* | - | JF295129.1 | *Biscogniauxia mediterranea* | 96/99 |  |
| **9** | HM559901.1 | *Uncultured fungus* | 97/99 | - | *-* | - | Colony light or yellow brown, with furry structure (6.2). |
|  | HM559873.1 | *Uncultured fungus* | 97/99 | - | *-* | - |  |
|  | EU593767.1 | *Xylaria sp.* | 97/99 | - | *-* | - |  |
| **10** | FJ556907.1 | *Cladosporium cladosporioides* | 97/99 | HQ443256.1 | *Cladosporium cladosporioides* | 99/99 | Colony black, with creamy structure. Furrows in agar (2.9). |
|  | AY897929.2 | *Uncultured organism* | 97/99 | JF793536.1 | *Cladosporium cladosporioides* | 99/99 |  |
|  | AY897945.1 | *Uncultured organism* | 97/99 | HQ671181.1 | *Cladosporium cladosporioides* | 99/100 |  |
| **11** | JQ390166.1 | *Phomopsis sp.* | 100/99 | GU584955.1 | *Phomopsis* sp. | 98/99 | Colony white-brown, with cottony structure. Dark-grey/brown spore accumulations (2.9) |
|  | AB665315.1 | *Phomopsis mali* | 100/99 | GU584957.1 | *Phomopsis* sp. | 98/99 |  |
|  | AB665312.1 | *Phomopsis sp.* | 100/99 | GU584956.1 | *Phomopsis* sp. | 98/99 |  |
| **12** | JQ686920.1 | *Chaetomium globosum* | 99/99 | AY681180.1 | *Asordaria sibutii* | 98/99 | Colony dark brown to black, with furry structure; partly light, with cottony structure. Black spore accumulations (13.3). |
|  | JQ067910.1 | *Remersonia thermophila* | 99/99 | HM122994.1 | *Fungal sp.* | 98/99 |  |
|  | JQ321261.1 | *Uncultured Sordariomycetes* | 99/99 | AY681171.1 | *Sordaria lappae* | 98/99 |  |
| **13** | AB277860.1 | *Isaria javanica* | 99/83 | JF720035.1 | Uncultured fungus | 97/99 | Colony white; both center and periphery furry, with a creamy zone in the middle (0.3). |
|  | AY198389.1 | *Coniochaeta ligniaria* | 93/98 | FJ228192.1 | *Lecythophora* sp. | 94/99 |  |
|  | AJ496242.1 | *Coniochaeta ligniaria* | 93/98 | HM123563.1 | Fungal sp. | 98/97 |  |
| **14** | JN546134.1 | *Apiospora montagnei* | 99/99 | HQ832955.1 | Fungal sp. | 100/99 | Colony light brown or yellow, with creamy structure or grey with furry structure. Black-grey spore accumulations (2.7). |
|  | JN397380.1 | *Arthrinium marii* | 99/99 | FJ462766.1 | *Arthrinium phaeospermum* | 99/99 |  |
|  | JN040736.1 | *Apiospora montagnei* | 99/99 | EF540751.1 | *Apiospora sp.* | 99/99 |  |
| **15** | EU167562.1 | *Bagnisiella examinans* | 97/100 | AY573207.1 | *Botryosphaeria sarmentorum* | 99/100 | Colony black-grey, with cottony structure. (8.4). |
|  | EF137358.1 | *Devriesia staurophora* | 97/100 | AY573206.1 | *Botryosphaeria sarmentorum* | 99/100 |  |
|  | EF137359.1 | *Devriesia staurophora* | 97/100 | EU673315.1 | *Diplodia acerina* | 99/99 |  |
| **16** | AB665311.1 | *Paraphaeosphaeria sp.* | 99/99 | EU552105.1 | *Camarosporium brabeji* | 100/99 | Colony center white/yellow, with cottony structure,  periphery yellow with creamy structure. White-yellow droplets (2). |
|  | AB534488.1 | *Uncultured fungus* | 99/99 | EU552104.1 | *Camarosporium brabeji* | 100/99 |  |
|  | HQ691430.1 | *Leptosphaeria coniothyrium* | 99/99 | JQ346215.1 | *Camarosporium brabeji* | 96/99 |  |
